# Microbial trimethylamine-*N*-oxide as a disease marker: something fishy?

**DOI:** 10.1080/16512235.2017.1327309

**Published:** 2017-05-19

**Authors:** Bjarne Landfald, Jørgen Valeur, Arnold Berstad, Jan Raa

**Affiliations:** ^a^ Norwegian College of Fishery Science, UiT The Arctic University of Norway, Tromsø, Norway; ^b^ Unger-Vetlesen Institute, Lovisenberg Diaconal Hospital, Oslo, Norway

**Keywords:** Carnitine, choline, gut microbiota, seafood, TMA, TMAO

## Abstract

Production of trimethylamine-*N*-oxide (TMAO) via the gut microbiota has recently been proposed as an important pathophysiological mechanism linking ingestion of ‘unhealthy foods’, such as beef (containing carnitine) and eggs (containing choline), and the development of atherosclerosis. Hence, TMAO has gained attention as a novel biomarker for cardiovascular disease. However, fish and seafood contain considerable amounts of TMAO and are generally accepted as cardioprotective: a puzzling paradox that seems to have been neglected. We suspect that the TMAO story may be a red herring.

Recently published works suggest a causal link between increased plasma levels of trimethylamine-*N*-oxide (TMAO) and increased risk of cardiovascular disease [1–3]. Plasma TMAO is produced enzymically [by flavin-containing monooxygenase-3 (FMO3)] in the liver by the oxidation of trimethylamine (TMA) generated by the gut microbiota. TMAO is an odourless compound, whereas TMA has the smell of spoiled fish. People with a defective FMO3 secrete TMA into their bodily fluids and thus attain a smell of spoiled fish, a condition designated ‘fish odour syndrome’ [4].

TMA is produced enzymically (by TMA-lyase) from the TMA-containing dietary components choline and carnitine by gut bacteria, and after uptake it is oxidized in the liver to TMAO and excreted. TMAO has been described as a toxic substance, and it has been suggested that broad-spectrum inhibitors of the bacterial TMA-lyase enzymes that generate TMA – the precursor of TMAO – may ‘revolutionize the preventive treatment’ of atherosclerotic cardiovascular disease [5]. The idea of enriching animal feeds with TMAO to improve their performance and growth [6] is, however, not in accordance with the assumption that TMAO is toxic.

According to the group of Hazen [3], there exists ‘a mechanistic link between intestinal microbe-dependent generation of trimethylamine-*N*-oxide (TMAO) and increased risk for future cardiovascular events (death, myocardial infarction, and stroke) by a pathway involving dietary nutrients such as phosphatidylcholine, choline, and carnitine’. Fish and other seafoods contain significant quantities of free TMAO, in addition to the TMA precursors (phosphatidylcholine, choline, and carnitine) found in meat, eggs, and milk products. The role of this free TMAO has apparently been neglected or overlooked in the discussion on the possible causal link between plasma TMAO and risk of cardiovascular disease. We recently raised this point in a Letter to the Editor of the *Journal of the American College of Cardiology* [7], which was commented upon by Tang and Hazen [8]. In the following, we extend and elaborate on our arguments.

Free TMAO in fish and marine invertebrates exerts important functions, which largely are associated with alleviating protein-denaturing effects of stressors such as high levels of inorganic ions or hydrostatic pressure. The molecule plays a particularly crucial role in cartilaginous fishes, where it counteracts the destabilizing effects of the high intracellular and extracellular concentrations of urea [9]. TMAO has also been associated with provision of buoyancy for groups of fish that lack a swimbladder [10]. In cod, a typical representative of a commercial teleost fish species, the TMAO level in muscle tissue has been reported to vary between 45 and 50 mmol/kg [11,12], making the molecule a significant contributor to the pool of cellular osmolytes. Moreover, the TMAO concentration tends to be several times higher than this level both in cartilaginous fish species and in deepwater teleosts [9,12]. By comparison, the total concentration of choline units (quarternary ammonium groups) in eggs is equivalent to 24 mmol/kg. In red meat, which has been in focus in the discussion on the association between plasma TMAO and the risk of cardiovascular diseases, the choline level amounts to less than 10 mmol/kg. The only foodstuff with a TMA-generating potential from choline equal to that of TMAO in marine fish species is liver from bovines and chickens [13]. In red meat, the concentration of carnitine amounts to approximately 6 mmol/kg, i.e. a little more than half of the total choline concentration. Other foods, including pork and fish, have levels of carnitine that are only a fraction of this [14]. Hence, free TMAO in seafood is quantitatively significantly higher than the amount of TMAO that can be generated by the gut microbiota from choline and carnitine in red meat and eggs.

Organ et al. [15] have shown, using mouse models, that TMAO added to feed (0.12%) resulted in a plasma level of TMAO equivalent to a 10 times higher admixture of choline. Moreover, TMAO reaches much higher levels in people on a seafood diet (> 5000 μmol/l) than those on an egg and red meat diet (139 μmol/l) [16]. Indeed, urinary TMAO may even be a biomarker for seafood consumption. In humans, the plasma level of TMAO increases rapidly after fish consumption [17], indicating direct uptake of free TMAO. It is not known, however, whether all free TMAO in fish is taken up directly or if some of it is transported into anaerobic compartments in the gut. Since TMAO is a preferred electron acceptor in anaerobic bacterial respiration [18], any free TMAO in the gut will inevitably be reduced to TMA by facultative anaerobic bacteria and thus become a source of TMA that comes in addition to that generated by TMA-lyase. Whether TMAO is taken up directly or as TMA after bacterial reduction of TMAO is, however, of subordinate importance for the evaluation of TMAO as a risk factor in atherosclerosis – seafood results undoubtedly in higher levels of plasma TMAO than intake of other foods.

If there should be any direct causal link between plasma TMAO and the risk of cardiovascular disease, it seems like a paradox that more fish in the diet reduces this risk [19,20]. Recent randomized group trials indicate that this effect goes beyond what can be associated with intake of marine lipids, since a diet with a large proportion of lean white fish (rich in TMAO) also reduces the risk [21]. There is, accordingly, an obvious need to clarify the significance of plasma or urinary TMAO as a disease marker, but it seems highly unlikely that free TMAO is the direct cause. Instead, increased risk of atherosclerosis may be associated with the abundance of TMA-producing bacteria in the gut, and hence indirectly with the TMAO generated by oxidation (i.e. detoxification) of TMA in the liver, as suggested also by Cho et al. [17]. The same authors showed that the ratio of Firmicutes to Bacteriodetes in the gut microbiota was notably higher in individuals producing high TMAO levels than in low TMAO producers, hence supporting the idea that the correlation between TMAO and atherosclerosis is indirect and likely to be the result of dysbiotic gut microbiota. The phenolic antioxidant resveratrol, often referred to as the anti-atherosclerosis principle in red wine, inhibits TMAO production by reduced TMA production after remodelling the gut microbiota [22].

Bacterial production of TMA from choline and carnitine is an entirely different biochemical process from the putative production of TMA from free dietary TMAO. In the latter case, TMAO will be an electron acceptor in oxidative respiration, with a high yield of energy for bacterial growth. In the former case, liberation of TMA from choline and carnitine does not generate energy for bacterial growth. However, the aldehydes produced in the cleavage reactions are energy-rich substrates in anaerobic fermentation reactions and in respiration. Bacteria producing choline TMA-lyase are apparently not very frequent in the healthy gut microbiota [23] and they are probably not the same as those able to use TMAO as an electron acceptor in energy-generating anaerobic respiration, and *vice versa*. Hence, TMA produced by the different pathways reflects different activities and composition of the gut microbiota. TMAO produced in the liver from TMA generated when bacteria consume choline and carnitine in the gut may, therefore, merely be a biomarker of the excessive presence of particular bacterial species that have become so abundant that the microbiota has become dysbiotic.

Optimism has been expressed regarding the prospect of using TMA-lyase inhibitors as new pharmaceutical drugs against cardiovascular disease [5]. The concept is to reduce TMAO production in the liver by inhibiting the production of precursor TMA in the gut. If, however, TMAO as such is a primary risk factor for atherosclerosis, then such drugs would be of little help for seafood lovers. They would have an ample supply of ‘toxic’ TMAO from the healthy food they eat. On the other hand, free TMAO from seafood may act as an electron acceptor for facultative anaerobic bacteria able to respire with the aldehydes resulting from the TMA elimination reactions of choline and carnitine. It may well be that TMAO-rich seafood would have the same effect – indirectly – as the TMA-lyase inhibitors, not by inhibiting TMA-lyase, but by supporting growth of more beneficial TMA-producing species. This is, of course, speculation, but seafood is, after all, also known to counteract negative effects of other foods.
